# β-catenin signaling inhibitors ICG-001 and C-82 improve fibrosis in preclinical models of endometriosis

**DOI:** 10.1038/s41598-019-56302-4

**Published:** 2019-12-27

**Authors:** Tomoko Hirakawa, Kaei Nasu, Saori Miyabe, Hiroyuki Kouji, Akira Katoh, Naoto Uemura, Hisashi Narahara

**Affiliations:** 1 Department of Obstetrics and Gynecology, Faculty of Medicine, Oita University, Oita, Japan; 2 Division of Obstetrics and Gynecology, Support System for Community Medicine, Faculty of Medicine, Oita University, Oita, Japan; 3 Translational Chemical Biology Laboratory, Faculty of Medicine, Oita University, Oita, Japan; 4 Department of Clinical Pharmacology and Therapeutics, Faculty of Medicine, Oita University, Oita, Japan

**Keywords:** Cell biology, Chemical biology, Drug discovery, Physiology, Diseases, Medical research, Molecular medicine, Oncology, Pathogenesis

## Abstract

Endometriosis exhibits unique characteristics, such as fibrosis, resistance to apoptosis, and promotion of cell proliferation; however, its pathophysiology is not fully understood. Recurrence rates after treatment are high, and the progression risk continues until menopause; hence, more effective therapy for endometriosis is needed. CREB-binding protein (CBP)/β-catenin signaling inhibitors have demonstrated antifibrogenetic effects in liver, lung, and skin diseases. The present study evaluated the effects of two CBP/β-catenin signaling inhibitors, ICG-001 and C-82, on the progression of endometriosis using endometriotic cyst stromal cells from the ovary and normal endometrial stromal cells from the uterus. ICG-001 was also evaluated in a mouse model. ICG-001 and C-82 inhibited cell proliferation, fibrogenesis, and cell migration, and promoted apoptosis *in vitro*. ICG-001 inhibited the growth of endometriotic lesions in the mouse model. CBP/β-catenin signaling plays an important role in the pathophysiology of endometriosis. Inhibiting the CBP/β-catenin signal can be a therapeutic target for endometriosis.

## Introduction

Endometriosis is a benign estrogen-dependent disease in which endometrial tissue develops outside the uterus, such as in the ovaries, peritoneum, and rectovaginal space. Most patients with endometriosis are of reproductive age. The main symptoms of endometriosis are dysmenorrhea, chronic pelvic pain, subfertility, and dyspareunia, which often decrease quality of life significantly^1^. Pathologically, endometriosis is diagnosed by the presence of endometrial glands, endometrial stroma, and hemosiderin laden macrophages outside the uterus, mainly in the ovaries. Although the pathophysiology of endometriosis is not fully understood, it is characterized by fibrosis (scarring), resistance to apoptosis, and promotion of cell proliferation^2^. Pain from endometriosis can be treated by excising the peritoneal lesions and ovarian cysts, or using hormonal agents such as progestin, oral contraceptives, and gonadotropin-releasing hormone agonists. However, these treatment strategies are associated with high recurrence rates^1^; hence, more effective therapy is needed.

There are some reports about the relationship between fibrosis and the progression of endometriosis^2,3^. We hypothesized that targeting fibrosis can be a new treatment strategy for endometriosis. Studies on fibrosis in endometriosis have reported a possible correlation between aberrant activation of the Wnt/β-catenin signaling pathway and progression of endometriosis^4,5^_._ The Wnt/β-catenin signaling pathway activates fibrosis and is associated with fibrotic diseases in lung, skin, kidney, and liver^6–8^. While drugs with sufficient antifibrotic activity have not yet been identified, PRI-724 (PRISM Pharma Co., Ltd., Kanagawa, Japan), a CBP/β-catenin-specific antagonist, is a promising antifibrotic drug against liver cirrhosis whose safety, tolerability, and antifibrotic effect were assessed in several patients with hepatitis C virus (HCV) cirrhosis^8–10^. An active metabolite of PRI-724 called C-82 was discovered from the early lead compound ICG-001, which binds to CBP and inhibits CBP/β-catenin binding^8,11^. ICG-001 was reported as an effective treatment for bleomycin-induced lung fibrosis in mice^12^.

Our study group has already validated a cell culture experimental assay with endometriotic cyst stromal cells (ECSC) from ovarian endometriomas (chocolate cysts) and normal endometrial stromal cells (NESC) from the uterus for chemical screening studies^13–15^. Therefore, in the present study, we evaluated ICG-001 and C-82 using ECSC and NESC in *in vitro* functional assays, and ICG-001 in *in vivo* experiments using an established mouse model of endometriosis.

## Results

### β-catenin expression

**Figure 1:**
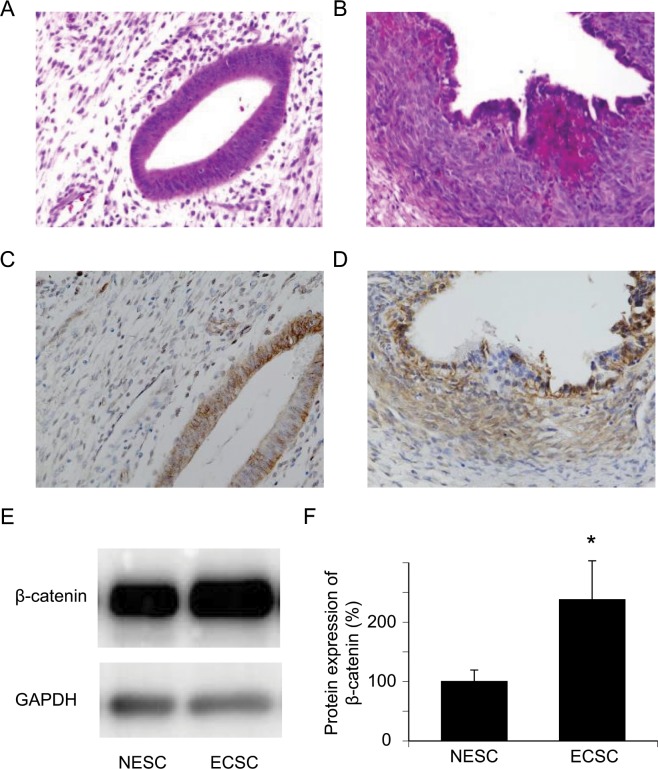
Expression of β-catenin is upregulated in endometriosis. (**A**) HE staining of a normal endometrium. An endometrial gland and endometrial stromal cells are shown. (**B**) HE staining of endometriosis. (**C**) A representative image of immunohistochemical staining of a normal endometrium with anti-human β-catenin. (**D**) A representative image of immunohistochemical staining of endometriosis with anti-human β-catenin. (**E,F**) Significant upregulation of β-catenin protein expression in ECSC compared with NESC is shown by western blotting. n = 5, *p < 0.01, Student’s *t*-test. Error bars represent standard deviation (SD). Uncropped images are shown in Fig. S5.

β-catenin expression in endometriotic lesions from patients with ovarian endometriosis (n = 5) as well as uterine endometrium from patients without endometriosis (n = 5) were evaluated by immunohistochemistry (Fig. 1). Figure 1A,B show hematoxylin and eosin (HE) stained normal endometrium and an endometriotic cyst of the ovary, respectively. The epithelial cells of both the normal endometrium (Fig. 1C) and endometriotic cyst (Fig. 1D) were stained almost equally and intensely with β-catenin. Although the stromal cells were stained partially and weakly, western blot analysis showed that β-catenin expression in ECSC was significantly higher than in NESC (Fig. 1E,F, Student’s t-test, p = 0.0020).

### Assessment of cell viability, cell proliferation and apoptosis

**Figure 2:**
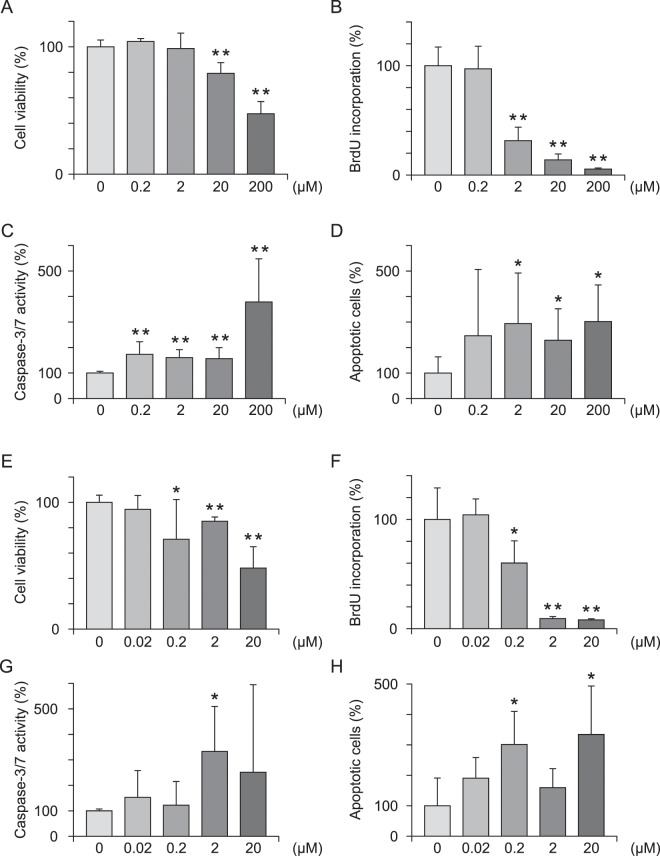
ICG-001 and C-82 inhibit cell proliferation and promote apoptosis in ECSC. (**A**) MTT assay with ICG-001. (**B**) BrdU assay with ICG-001. (**C**) Caspase 3/7 assay with ICG-001. (**D**) Cell death detection ELISA with ICG-001. (**E**) MTT assay with C-82. (**F**) BrdU assay with C-82. (**G**) Caspase 3/7 assay with C-82. (**H**) Cell death detection ELISA with C-82. n = 6. *p < 0.05, **p < 0.001, Student’s *t*-test. Error bars represent standard deviation (SD).

We next examined cell proliferation and apoptosis in ECSC after treatment with CBP/β-catenin signaling inhibitors. ICG-001 significantly inhibited cell viability by 20.8% and 52.6% compared with the control at concentrations of 20 and 200 µM, respectively, in the methylthiazoletetrazolium (MTT) assay (Fig. 2A, p = 0.1178, p = 0.8090, p = 0.0005, p = 0.0000), and inhibited cell proliferation by 68.6%, 86.1%, 94.5% at concentrations of 2–200 µM, respectively, in the 5-bromo-2′-deoxyuridine (BrdU) assay (Fig. 2B, p = 0.8900, p = 0.0000, p = 0.0000, p = 0.0000). Furthermore, ICG-001 increased apoptosis by 73%, 60.6%, 56.4%, and 278.7% at concentrations of 0.2–200 µM, respectively, in the Caspase 3/7 assay (Fig. 2C, p = 0.0050, p = 0.0008, p = 0.0010, p = 0.0024), and by 194.3%, 128.9%, and 202.3% at 2–200 µM, respectively, in the cell death detection ELISA (Fig. 2D, p = 0.2107, p = 0.0453, p = 0.0463, p = 0.0103). C-82 inhibited cell viability by 29.1%, 14.9%, and 51.8% at concentrations of 0.2–20 µM, respectively, in the methylthiazoletetrazolium (MTT) assay (Fig. 2E, p = 0.3042, p = 0.0491, p = 0.0003, p < 0.0001), and inhibited cell proliferation by 39.9%, 90.7%, and 91.9% at concentrations of 0.2–20 µM, respectively, in the 5-bromo-2′-deoxyuridine (BrdU) assay (Fig. 2F, p = 0.7544, p = 0.0197, p < 0.0000, p < 0.0000), and increased apoptosis by 233% at 2 µM in the Caspase 3/7 assay (Fig. 2G, p = 0.24808, p = 0.5742, p = 0.0090, p = 0.3060), and by 201.7% and 234.2% at 0.2 and 20 µM, respectively, in the cell death detection ELISA (Fig. 2H, p = 0.0781, p = 0.0058, p = 0.2179, p = 0.0108).

### Scratch assay

**Figure 3:**
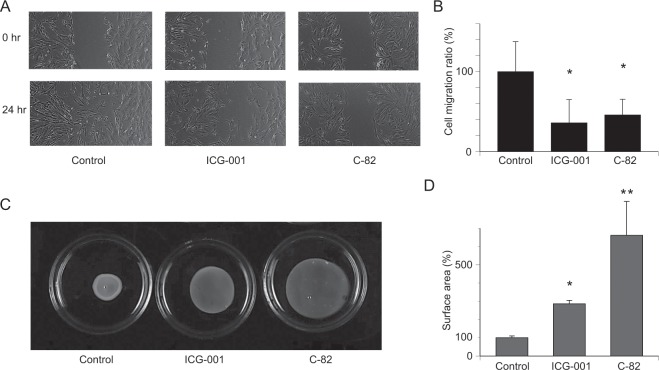
Cell migration and fibrosis are significantly inhibited by ICG-001 and C-82. (**A**) Scratch assay for 24 hours. Representative photos at 0 and 24 hours are shown. The control became confluent after 24 hours. (**B**) The analysis of the reduction of the ratio of the scratched area (cell migration ratio) at 24 hours. The data are shown as relative values at 24 hours against the scratched area of the controls at 0 hours. ICG-001 and C-82 inhibited cell migration significantly compared with controls. n = 5. *p < 0.05, Student’s *t*-test. (**C**) Representative result of a collagen gel contraction assay in ECSC. The concentrations of ICG-001 and C-82 were 20 μM and 2 μM, respectively. (**D**) Collagen gel contraction was significantly inhibited by ICG-001 and C-82. n = 3. *p < 0.001, **p < 0.01, Student’s *t*-test. Error bars represent standard deviation (SD).

In a scratch assay to evaluate cell migration (Fig. 3A), the untreated control ECSC migrated and became confluent 24 hours after the scratch. ICG-001 and C-82 inhibited cell migration (Fig. 3A). Migration distances were calculated and defined as 100% in the control group. Figure 3B shows a significant decrease in cell migration by 64% with ICG-001 and by 54% with C-82 in ECSC (p = 0.0168, p = 0.0212).

### Gel contractility

Three-dimensional collagen gel cultures of ECSC after treatment with the CBP/β-catenin signaling inhibitors showed significantly inhibited contractility by ICG-001 and C-82 as compared with the untreated control. The surface area was calculated and defined 100% in the control group. The ICG-001 group was 286% and the C-82 group was 662% in ECSC (Fig. 3C,D, p = 0.0001, p = 0.0063).

### α-SMA expression

**Figure 4:**
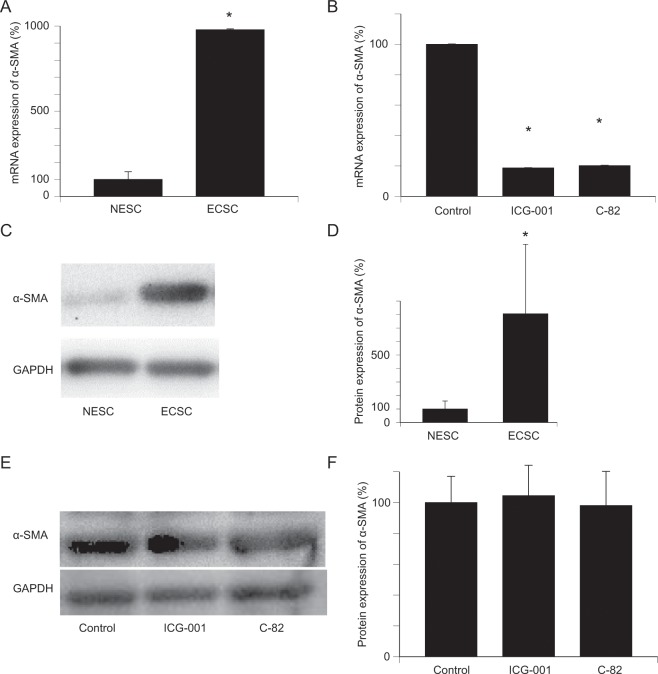
Expression of α-SMA is significantly upregulated in ECSC, and downregulated by ICG-001 and C-82. (**A**) The mRNA expression of α-SΜΑ was significantly upregulated in ECSC compared with NESC. n = 4. *p < 0.01, Student’s *t*-test. (**B**) mRNA expression of α-SΜΑ in ECSC was significantly downregulated by ICG-001 and C-82. n = 4. *p < 0.001, Student’s *t*-test. (**C,D**) Western blot analysis of α-SMA showed that protein expression of α-SMA was significantly upregulated in ECSC compared with NESC. n = 3. *p < 0.01, Student’s *t*-test. (**E,F**) Western blot analysis of α-SMA in control, ICG-001, and C-82 groups. There was no significant difference in the protein expression of α-SMA between the untreated control group and CBP/β-catenin inhibitor-treated groups by Student’s *t*-test. n = 4. Error bars represen*t* standard deviation (SD). Uncropped images are shown in Figs. S6 and S7.

The expression of alpha-smooth muscle actin (α-SMA) mRNA was significantly higher in ECSC than in NESC (Fig. 4A, p = 0.0018). Treatment with ICG-001 and C-82 significantly downregulated α-SMA mRNA expression in ECSC (Fig. 4B, p = 0.0000, p = 0.0000). The protein expression of α-SMA as examined by western blot analysis was significantly higher in ECSC than in NESC (Fig. 4C,D, p = 0.0400), but neither ICG-001 nor C-82 resulted in a significant change in the protein expression of α-SMA in ECSC (Fig. 4E,F, p = 0.7381, p = 0.8958).

### Endometriosis in mice model

**Figure 5:**
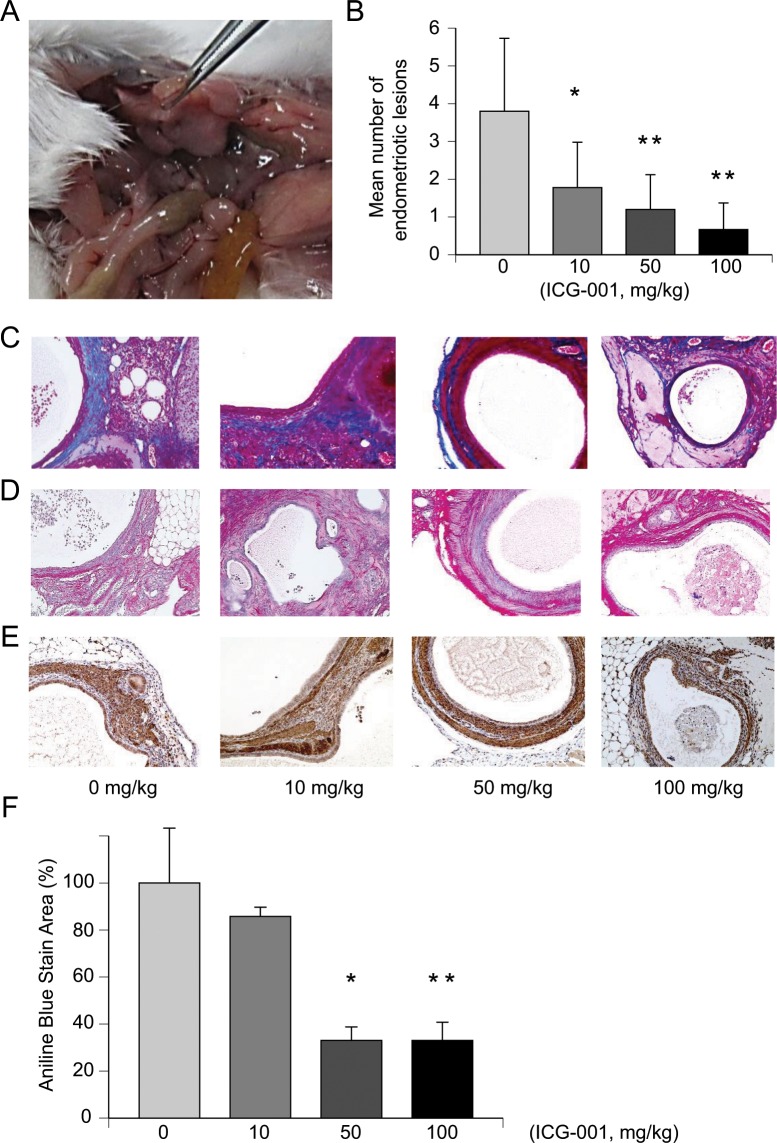
ICG-001 inhibits endometriosis progression in mice. (**A**) Representative abdominal findings. Endometriotic lesions are mainly observed as small cysts. (**B**) The mean number of endometriotic lesions in each ICG-001-treated group was significantly smaller than in the untreated control group. n = 10, 9, 10, and 9, for 0 mg/kg ICG-001, 10 mg/kg ICG-001, 50 mg/kg ICG-001, and 100 mg/kg ICG-001, respectively. *p < 0.01, **p < 0.001, Bonferroni correction. (**C**) Collagen fibers appear blue (Aniline blue) in modified Masson’s staining. (**D**) Collagen fibers appear pink in Sirius red staining. (**E**) Immunohistochemical staining with anti-α-SMA. (**F**) Analysis of the stain area of collagen fibers in modified Masson’s staining. The percentage of the Aniline blue area was significantly decreased in the 50-mg/kg ICG-001 and 100-mg/kg ICG-001 groups. n = 3. *p < 0.01, **p < 0.001, Bonferroni correction. Error bars represent standard deviation (SD).

In the experiments using a mouse model of endometriosis, we first confirmed endometriotic lesions having been formed in the mouse abdomen 1 week after uterine implantation (Table S1) and then evaluated the therapeutic effects of ICG-001 in these mice (n = 10/group, x 4 groups). One mouse in the ICG-001 10 mg/kg group and one mouse in the ICG-001 100 mg/kg group died about 3 weeks after uterine implantation. After examining the mice by dissection, the cause of death was assumed to be excess bleeding caused by injections. The weights of the mice in the four groups were not significantly different (Fig. S1). All groups were confirmed to have endometriotic lesions by HE staining (Fig. S2A–D). Figure 5A shows a representative picture of an intraabdominal endometriotic lesion. The mean number of endometriotic lesions in the untreated group was significantly higher than those in all the ICG-001-treated groups (Fig. 5B, p = 0.0102, p < 0.001, p < 0.001). The total weight of the endometriotic lesions was highest in the untreated group and lowest in the ICG-001 100 mg/kg group (Fig. S3). The fibrosis of endometriosis was assessed immunohistochemically by modified Masson’s staining (Fig. 5C), Sirius red staining (Fig. 5D), and α-SMA staining (Fig. 5E). We calculated the intensity of the collagen fibers in the modified Masson’s staining using a Keyence BZ-9000 (Keyence, Chicago, IL, USA). The collagen fibers (aniline blue stained area) were significantly reduced in the ICG-001 50 mg/kg and 100 mg/kg groups (Fig. 5F, p = 0.0012, p < 0.001). α-SMA was observed in endometrial stromal lesions and its staining intensity was the weakest in the ICG-001 100 mg/kg group (Fig. 5E).

## Discussion

The Wnt/β-catenin signaling pathway is involved in normal reproduction and development, as well as maintenance of normal physiological functions. The Wnt/β-catenin signaling pathway is further involved in progression of diseases by regulating cell proliferation, migration, invasion, and fibrogenesis^11,12,16,17^. Wnt/β-catenin signaling pathway inhibitors have been studied as potential treatments for various proliferative or fibrotic diseases, including cancers, liver cirrhosis or pulmonary fibrosis, and some inhibitors are currently being tested in clinical trials^8,17^. Although the antifibrotic effects of CBP/β-catenin inhibitors on lung fibrosis, liver fibrosis, and cancers have been examined^6,9,11,18,19^, their antifibrotic effect in endometriosis have not been investigated. Because activation of the Wnt/β-catenin signaling pathway in endometriosis is associated with fibrosis, cell proliferation, and resistance to apoptosis^5,20–22^, the inhibition of this pathway is recognized as a promising strategy for treating endometriosis. ICG-001 is a selective low molecular-weight inhibitor, and downregulates the expression of a subset of β-catenin/TCF-responsive genes^11^. ICG-001 inhibits β-catenin/TCF signaling by specifically binding to the cyclic AMP response element-binding protein CREBBP (CBP), thereby disrupting the CBP/catenin interaction^23^. PRI-724 is a second generation specific CBP/catenin antagonist^24^.

In this study, we tested a hypothesis that Wnt/β-catenin signaling inhibitors ICG-001 and C-82 have an antifibrotic effect in the preclinical models of endometriosis that had been validated by our group for evaluation of fibrosis^13,14,15^. The present study revealed significant upregulation of β-catenin protein expression in ECSC, suggesting activation of Wnt/β-catenin signaling pathway in the pathophysiology of endometriosis. ICG-001 and C-82 inhibited *in vitro* cell growth, cell migration, and fibrosis, and promoted apoptosis. The mRNA expression of α-SMA with ICG-001 and C-82 was significantly downregulated in ECSC, but there was no difference in protein expression (Fig. 4BE,F). It is believed that there is a time lag between the expression of mRNA and the expression of the protein. It is not expected that induced transcription immediately leads to increased protein levels because there is a delay to mature, export, and translate mRNA^25^.

Treatment with ICG-001 (10, 50, and 100 mg/kg) appeared to be nontoxic to mice, but effectively modified disease progression in the murine model of endometriosis where ICG-001 dose-dependently inhibited intra-abdomen fibrosis as evaluated. However, the number of endometriotic lesions, intensity of the modified Masson’s staining, and the staining intensity of α-SMA were the weakest in the ICG-001 100 mg/kg group. Thus, further study is needed. Data generated from this study provide additional evidence to support the inhibition of the Wnt/β-catenin signaling pathway being an attractive therapeutic target to improve fibrosis and to reverse endometriotic lesions.

Prior to the present study, the relationship between CBP/β-catenin binding and endometriosis had not been fully examined, although there are some reports illustrating that the Wnt/β-catenin pathway is activated in endometriosis. Matsuzaki *et al*. demonstrated that fibrosis, cell proliferation, and cell migration were inhibited in ECSC^5,22,26^ after blocking the Wnt/β-catenin pathway. The antifibrotic effect of CGP049040, a small molecule TCF/β-catenin antagonist, on fibrosis was shown in their mouse model of endometriosis. The staining scores by Sirius red and Masson trichrome stains were significantly lower in CGP049040 treated mice. Their data support our view that activation of the Wnt/β-catenin signaling pathway in endometriosis is associated with fibrosis. Although Matsuzaki *et al*. showed a reduction of collagen fiber by a small molecule antagonist for the TCF/β-catenin complex, they did not provide data on the reduction in size or the number of endometriotic lesions, whereas our study clearly demonstrated a reduction of endometriotic lesions in size and number with CBP/β-catenin inhibitors. CBP/β-catenin interaction is crucial in the pathological mechanism of endometriosis progression.

There are some limitations in the current study. Although no apparent drug-related toxicity was observed from the intraperitoneal findings or body weight, the effects on other organs have not been confirmed. There is no denying that this drug may affect the functions or morphology of other organs. PRI-724 has been administrated to humans, and a clinical trial of PRI-724 (C-82) revealed that most of the observed side effects to humans were mild, such as reaction at the injection site, nausea, and constipation^8^. A high dose of ICG-001 was given intraperitoneally, and this dose may not be comparable to the clinical dose. In this study, the pharmacokinetics of ICG-001 were not evaluated; therefore, it is necessary to investigate the relationship between exposure and pharmacodynamics. The present endometriosis model was created by transplanting the uterine tissue of a normal mouse into the abdominal cavity of another mouse. It may not accurately reflect the condition of human endometriosis and its translationability is unknown. The menstrual cycle was different from what naturally occurs in the model mice because estradiol was continuously administered. Although this was translational research at the stage of confirming safety, there is a history of mice models being widely used for endometriosis studies in general, and it has been validated as a model that shows the dynamics of endometriosis.

In conclusion, we have identified the CBP/β-catenin signaling inhibitors, ICG-001 and C-82, as leads to promising therapeutic agents for the treatment of endometriosis. The CBP/β-catenin inhibitors suppressed cell migration and cell proliferation and promoted apoptosis. Hence, our data suggest that CBP/β-catenin inhibitors have a possibility not only to prevent but reduce fibrosis in endometriosis. Future studies will need to focus on searching and synthesizing more effective CBP/β-catenin inhibitors. The promotion of research and practical application of new drugs for endometriosis is important, and a clinical trial of CBP/β-catenin inhibitors for endometriosis should be planned for the near future.

## Methods

### Cell culture and reagents

Ovarian endometriosis tissues were obtained from patients with regular menstrual cycles who had undergone salpingo-oophorectomy or cystectomy for the treatment of ovarian endometriotic cysts (11 patients, aged 26–49 years). Normal endometrium tissues were obtained from patients who had undergone hysterectomy because of myoma or other benign diseases without endometriosis (6 patients, aged 43–45 years). None of the patients had received hormonal treatments for at least 6 months before surgery. All specimens were confirmed to be in the proliferative phases based on pathological observation, the menstrual cycle, or both. ECSC and NESC were isolated from the ovarian endometriotic tissues and normal uterine tissues using enzymatic digestion with collagenase. They were then confirmed to be stromal cells from the endometrium as previously described^27,28,29^. In addition, we performed basic experiments using western blotting. CD10 and vimentin were positive, and we confirmed the cells were derived from endometrial stroma. Isolated cells were cultured in Dulbecco’s modified Eagle’s medium (DMEM, Nissui, Tokyo, Japan) supplemented with 100 IU/mL penicillin (Meiji Seika, Tokyo, Japan), 50 mg/mL streptomycin (Meiji Seika, Tokyo, Japan), and 10% heat-inactivated fetal bovine serum (FBS, all obtained from EQUITECH-BIO, Inc., Kerrville, Texas, USA) at 37 °C in air containing with 5% CO_2_. ECSC and NESC were confirmed to have >99% purity by immunocytochemical staining with vimentin (Santa Cruz Biotechnology, Dallas, USA), CD10 (Abcam, Cambridge, UK), cytokeratin (Bioss, Massachusetts, USA), and factor VIII (Novus Biologicals, Colorado, USA) after two to three passages. Cells from two to five passages were used. ICG-001 was synthesized in our laboratory and C-82 was provided by Prism Pharma Co., Ltd., Tokyo, Japan (Fig. S4). We have data that C-82 inhibits the binding between β-catenin and CBP 10 times more than ICG-001. The concentration used for acute lymphoblastic leukemia^23^ was the reference for this study.

This study was approved by the Institutional Review Board of the Faculty of Medicine, Oita University (no. P16–01), and written informed consent was obtained from all patients. All methods involving patients were performed in accordance with relevant guidelines and regulations.

### Immunohistochemical staining

Immunohistochemical staining (using the streptavidin–biotin–peroxidase method) was performed with mouse monoclonal antibody against human β-catenin (1:100 dilution; Cell Signaling #8480, Massachusetts, USA), and biotinylated goat anti-rabbit IgG (Dako, Tokyo, Japan). The staining intensities and sites of β-catenin in the endometriotic cysts of the ovaries compared with normal endometrial tissue were assessed.

### Assessment of protein expression

The protein levels of β-catenin and α-SMA in ECSC and NESC were evaluated by western blot analysis. Antibodies against β-catenin (Cell Signaling, MA, USA), α-SMA (Gene Tex, Irvine, California, USA), and GAPDH (FUJIFILM Wako Chemical Corporation, Osaka, Japan) were used. Total protein from ECSC cells that were treated with ICG-001 (20 µM) or C-82 (2 µM) for 48 hours was extracted. The expression of each protein relative to the GAPDH protein in ECSC and NESC was analyzed using Image Lab software (Bio-Rad Laboratories, Hercules, CA, USA). The data were presented as the percentage of the values obtained for ECSC compared with NESC.

### Assessment of cell viability

The viability of ECSC after ICG-001 or C-82 treatment was determined by a modified MTT assay using Cell Proliferation Kit I (Roche Diagnostics, Basel, Switzerland) as Hirakawa *et al*.^2^ reported previously. Briefly, 5 × 10^3^ ECSC were seeded into each well of a 96-well flat-bottom microplate (Corning, New York, NY, USA). After 24 hours, the culture medium was replaced with medium containing ICG-001 (0, 0.2, 2, 20, and 200 µM) or C-82 (0, 0.02, 0.2, 2, and 20 µM), and the cells were incubated for 24 hours. After that, 20 µL of the MTT reagent was added to each well, the cells were incubated for 4 hours, then 100 µL of detergent reagent was added to each well. Cell viability was determined by measuring the absorbance at 570 nm. The values obtained from the ICG-001 or C-82 treated ECSC are presented as a percentage of the values from untreated ECSC.

### Assessment of cell proliferation

The proliferation of ECSC following treatment with ICG-001 or C-82 was determined based on BrdU incorporation using an ELISA kit (Cell Proliferation ELISA, Roche Diagnostics, Basel, Switzerland) as Hirakawa *et al*.^2^ reported. ECSC (5 × 10^3^) were seeded into each well of a 96-well flat-bottom microplate (Corning 96-well, Corning, NY, USA). After 24 hours, the culture medium was replaced with medium containing ICG-001 (0, 0.2, 2, 20, and 200 µM) or C-82 (0, 0.02, 0.2, 2, and 20 µM), and the cells were incubated for 24 hours. Then, 10 µL BrdU (10 mM) was added to each well, followed by incubation for 2 hours. The incorporation of BrdU was determined by measuring the absorbance of the resultant solution at 450 nm. The values of the ICG-001 or C-82 treated ECSC are presented as a percentage of the values of untreated ECSC.

### Assessment of apoptosis

We determined the apoptosis levels of ECSC following ICG-001 or C-82 treatment by direct determination of nucleosomal DNA fragmentation using ELISA (Cell Death Detection ELISA, Roche Diagnostics, Basel, Switzerland) as Hirakawa *et al*.^2^ described. ECSC (5 × 10^3^ cells/well) were seeded in a 96-well flat-bottom microplate (Corning 96-well, Corning, NY, USA). After 24 hours, the culture medium was replaced with medium containing ICG-001 (0, 0.2, 2, 20, and 200 µM) or C-82 (0, 0.02, 0.2, 2, and 20 µM), and the cells were incubated for 24 hours. Then, the cells were lysed and centrifuged at 200 × *g* for 10 minutes, and the mono- and oligo-nucleosomes in the supernatants were quantified using an antihistone–biotin antibody (Nichirei Biosciences Inc., Tokyo, Japan). The concentration of the nucleosome–antibody complex was determined by measuring the absorbance at 405 nm using 2,2′-azino-di(3-ethylbenzthiazolinesulfonate) as the substrate. The values of ECSC treated with ICG-001 or C-82 are presented as a percentage of the values from untreated ECSC.

### Assessment of caspase-3 and caspase-7 activity

The caspase-3 and caspase-7 activity of ECSC following incubation with ICG-001 or C-82 was evaluated using the Caspase-Glo 3/7 assay (Promega, Madison, WI, USA) as Hirakawa *et al*.^2^ reported. ECSC (5 × 10^3^ cells/well) were seeded in a 96-well flat-bottom microplate. After 24 hours, the culture medium was replaced with medium containing ICG-001 (0, 0.2, 2, 20, and 200 µM) or C-82 (0, 0.02, 0.2, 2, 20 µM), and the cells were incubated for 24 hours. The supernatant of the medium and the Caspase-Glo 3/7 reagent were mixed in another 96-well flat-bottom microplate, the plate was shaken gently at 20–25 °C for 180 minutes, then the luminescence was measured using a plate-reading luminometer (GloMax, Promega, Madison, WI, USA). The values of ECSC treated with ICG-001 or C-82 are presented as a percentage of the values of untreated ECSC.

### Assessment of cell migration

The scratch assay was used to assess cell migration and proliferation. ECSC were incubated in 12-well culture plates. When cells were 80% confluent, each plate was scratched with the same straight line. The medium was replaced with medium containing ICG-001 (20 µM) or C-82 (2 µM). The concentrations were determined from the results of the cell proliferation assay and the assay of the anti-apoptotic effect. Photos were taken on a microscope and the gap distances were quantitatively evaluated using BZ9000 (Keyence Corp., Osaka, Japan) at 0 and 24 hours after the scratch. Narrow distance between scratch edges means great migration ability and strong cell growth. The ratio before and after migration was calculated. A migration ratio of 100% indicates the highest migration ability.

### Assessment of ECSC contractility

We have established a three-dimensional collagen gel culture system with ECSC as a model of fibrosis formation in endometriosis^2,29^. In this system, ECSC are cultured in floating collagen lattices to induce the reorganization and compaction of collagen fibers, resulting in the contraction of collagen gels. This culture system provides models of mechanically relaxed tissue with low tensile strength and the early developmental stage of endometriotic lesions with high tensile strength. Collagen gel contraction assays were performed as previously described^29^. Forty-eight hours after ICG-001 (20 µM) or C-82 (2 µM) treatment, the ECSC were embedded in the collagen gel (Cellmatrix type I-A; Nitta Gelatin, Osaka, Japan) and cultured three-dimensionally for a further 72 hours. ICG-001 (20 µM) or C-82 (2 µM) was also added to the culture medium. The collagen gels were then photographed and the area of the gel surface was measured with ChemiDoc XRS+ with Image Lab Software (Bio-Rad Laboratories, Hercules, California, USA).

### Assessment of mRNA expression

Total RNA of ECSC and NESC was extracted using a miRNeasy Mini kit (Qiagen, Düsseldorf, Germany) following the manufacturer’s instructions. In addition, total RNA from ECSC that were treated with ICG-001 (20 µM) or C-82 (2 µM) for 48 hours was extracted similarly to analyze the expression of α-SMA. Subsequently, cDNA was synthesized from 1 μg of total RNA using the Reverse Transcription System (Promega, Madison, WI, USA). Quantitative RT-PCR was carried out with a LightCycler 480 (Roche Diagnostics, Rotkreuz, Schweiz) using TaqMan Universal PCR Master Mix II with the following specific primers (Applied Biosystems, Thermo Fisher Scientific, Waltham, MA, USA): β-catenin (Assay ID: Hs00355045_m1); α-SMA (Assay ID: Hs04406862_m1); glyceraldehyde 3-phosphate dehydrogenase (GAPDH) (Assay ID: Hs02786624_g1) as Hirakawa *et al*.^2^ reported. The expression level of mRNA relative to GAPDH mRNA was calculated by the relative standard curve method. Standard curves for relative quantification were prepared. Quantity is expressed relative to an untreated control. The data from ECSC were presented as the percentage of the values compared with NESC.

### Mouse experiments

Before surgery, 40 8-week-old adult female mice (Balb/C, Charles River Laboratories, Wilmington, MA, USA) were habituated for 2 weeks at a controlled temperature (22–23 °C) on a 12-hour light, 12-hour dark cycle, and given food and water freely. The mice were divided into four groups: control group (ICG-001 0 mg/kg), ICG-001 10 mg/kg group, ICG-001 50 mg/kg group, and ICG-001 100 mg/kg group. The dosages were taken from previous studies^9,23^.

In addition, three mice were examined for endometriotic lesions 1 week after implantation, and an additional 44 mice were prepared as uterus donors. Mice were anesthetized with 2% isoflurane and, after bilateral dorsal incision, the bilateral ovaries were resected. The incision was sutured with 4–0 nylon. Estradiol (1 μg/mouse, Fuji Pharma, Tokyo, Japan, diluted with corn oil) was injected intraperitoneally into the left side of the abdomen once a week after the ovariectomy^30,31^. Two weeks after the ovariectomy, donor mice were euthanized, the uterus was removed, minced, and injected peritoneally with Corning Matrigel into the recipient mice. The uterus from each donor mouse was divided into two parts and implanted into two recipient mice. One week after uterine implantation, the sites and numbers of endometriotic lesions in the abdomen were examined. After confirming implantation of endometriosis, we started drug administration. Intraperitoneal injections (ICG-001 0, 10, 50, 100 mg/kg, diluted with PBS) to the right side of the abdomen were administered three times a week for 1 week after the implantation. Six weeks after ICG-001 administration, mice were euthanized and dissected to examine the abdomen. The sites of implantation and the numbers and weights of endometriotic lesions were recorded. An experimental assistant who was blinded to this experiment measured each resected specimen with an electronic analytical scale (SHIMADZU AG 204). Resected endometriotic lesions and uteruses were embedded in paraffin and stained by HE and anti-α-SMA antibody (Gene Tex, Irvine, California, USA). The histological tissue of the endometriotic lesions was also observed with modified Masson’s staining (Scy Tek Laboratories, Utah, USA) and Sirius red staining (Polysciences, Inc., PA, USA) to evaluate fibrosis. To assess the extent of fibrosis, we analyzed the stained areas of collagen fiber by modified Masson’s staining using BZ9000 (Keyence Corp., Osaka, Japan). The analysis area was defined by the detection of both endometrial epithelial cells and endometrial stromal cells under high-power magnification.

### Statistics

The data were analyzed and presented as percentages relative to the corresponding control values as mean ± standard deviation. All statistical analyses were performed using the Excel statistical software package (BellCurve for Excel, version 2.15; Social Survey Research Information Co., Ltd., Tokyo, Japan). Significance was set at p < 0.05 with the Bonferroni correction and Student’s *t*-test.

### Study approval

This study was approved by the Institutional Review Board of the Faculty of Medicine, Oita University (No. P16-01), and written informed consent was obtained from all patients. All methods involving humans were performed in accordance with relevant guidelines and regulations. The mouse model study was approved by Oita University Animal Ethics Committee (No. 172901). We followed the ARRIVE (Animal Research: Reporting of *In Vivo* Experiments) guidelines.

## Supplementary information

Supporting information

Supporting information 2

Supporting information 3

Supporting information 4

Supporting information 5

Supporting information 6
