# Supplementary material for: β-catenin signaling inhibitors ICG-001 and C-82 improve fibrosis in preclinical models of endometriosis
Source: Sci Rep. 2019 Dec 27;9:20056. doi: 10.1038/s41598-019-56302-4 (PMC6934788; doi:10.1038/s41598-019-56302-4)
Supplement: Supplementary file 1 — Supporting information [file 41598_2019_56302_MOESM1_ESM.docx]

Supplemental Table 1. Endometriotic lesion in Mice after a week from implantation.

| Number of mice | 3 |
| --- | --- |
| Mean number of endometriotic lesions (range) | 2.667 (2-3) |
| Mean weight of endometriotic lesions (range) -g | 0.1676 (0.0368-0.3812) |
| Mean weight of mouse (range) -g | 14.39 (13.75-15.25) |
