# Supplementary figures and images for: β-catenin signaling inhibitors ICG-001 and C-82 improve fibrosis in preclinical models of endometriosis
Source: Sci Rep. 2019 Dec 27;9:20056. doi: 10.1038/s41598-019-56302-4 (PMC6934788; doi:10.1038/s41598-019-56302-4)

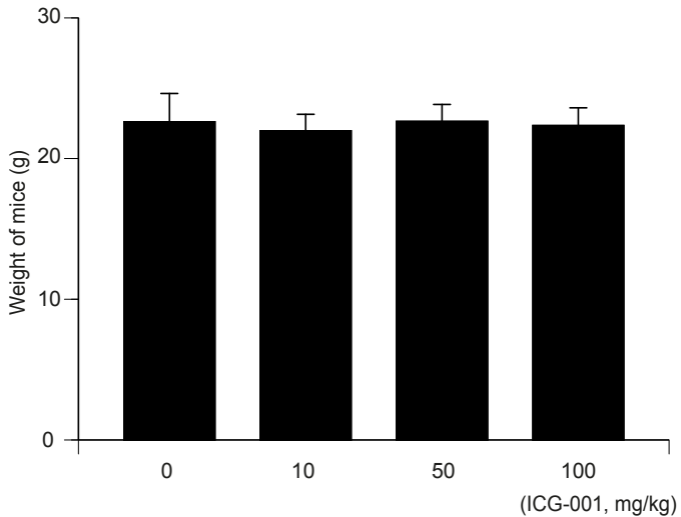

Supplement: Supplementary file 2 — Supporting information 2 [file 41598_2019_56302_MOESM2_ESM.pdf]

A

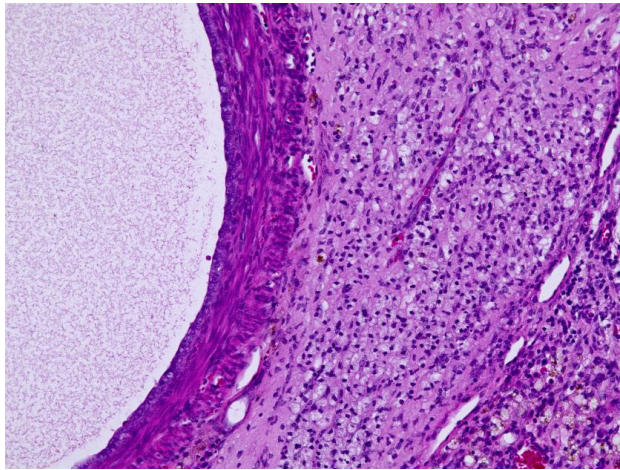

B

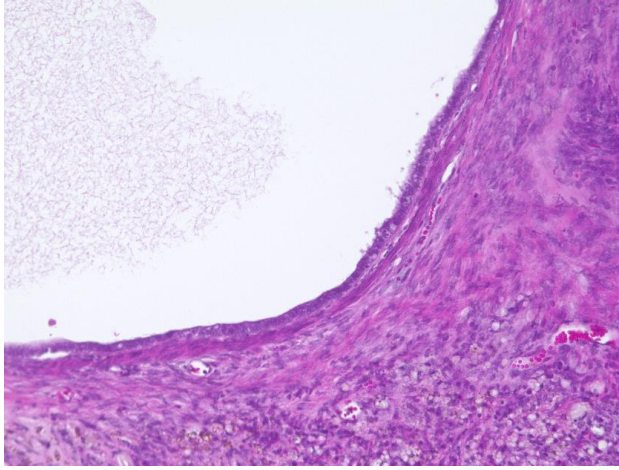

C

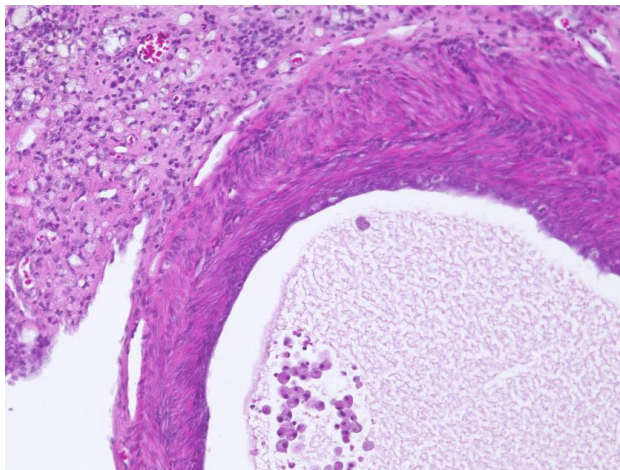

D

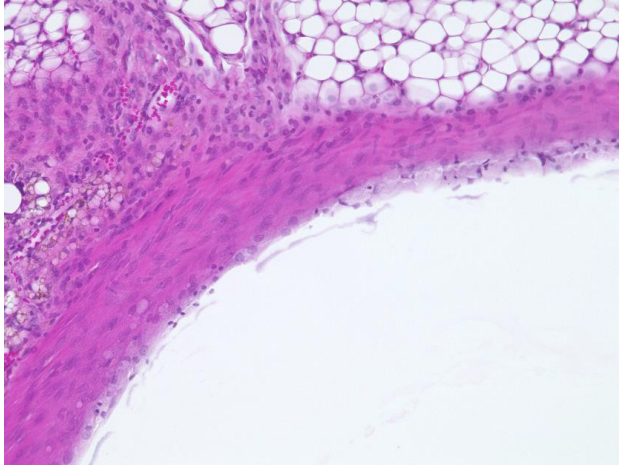

Supplement: Supplementary file 3 — Supporting information 3 [file 41598_2019_56302_MOESM3_ESM.pdf]

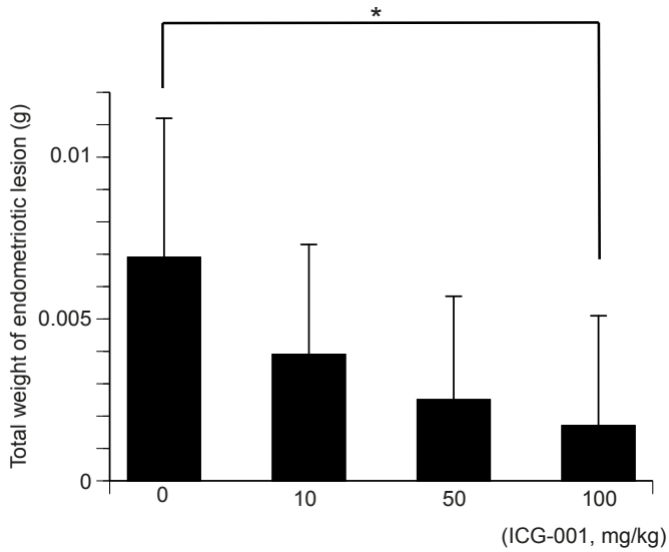

Supplement: Supplementary file 4 — Supporting information 4 [file 41598_2019_56302_MOESM4_ESM.pdf]

ICG-001

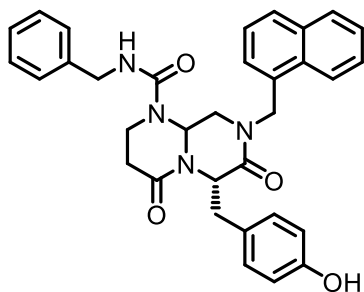

Chemical Formula:  $C_{33}H_{32}N_4O_4$   
Molecular Weight: 548.64

C-82

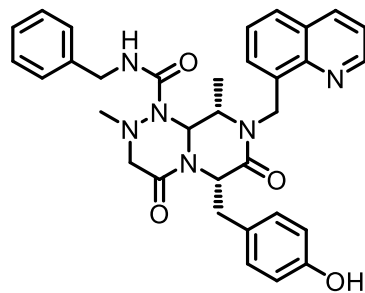

Chemical Formula:  $C_{33}H_{34}N_6O_4$   
Molecular Weight: 578.67

Supplement: Supplementary file 5 — Supporting information 5 [file 41598_2019_56302_MOESM5_ESM.pdf]
