# Supplementary material for: β-catenin signaling inhibitors ICG-001 and C-82 improve fibrosis in preclinical models of endometriosis
Source: Sci Rep. 2019 Dec 27;9:20056. doi: 10.1038/s41598-019-56302-4 (PMC6934788; doi:10.1038/s41598-019-56302-4)
Supplement: Supplementary file 6 — Supporting information 6 [file 41598_2019_56302_MOESM6_ESM.pptx]

## Slide 1
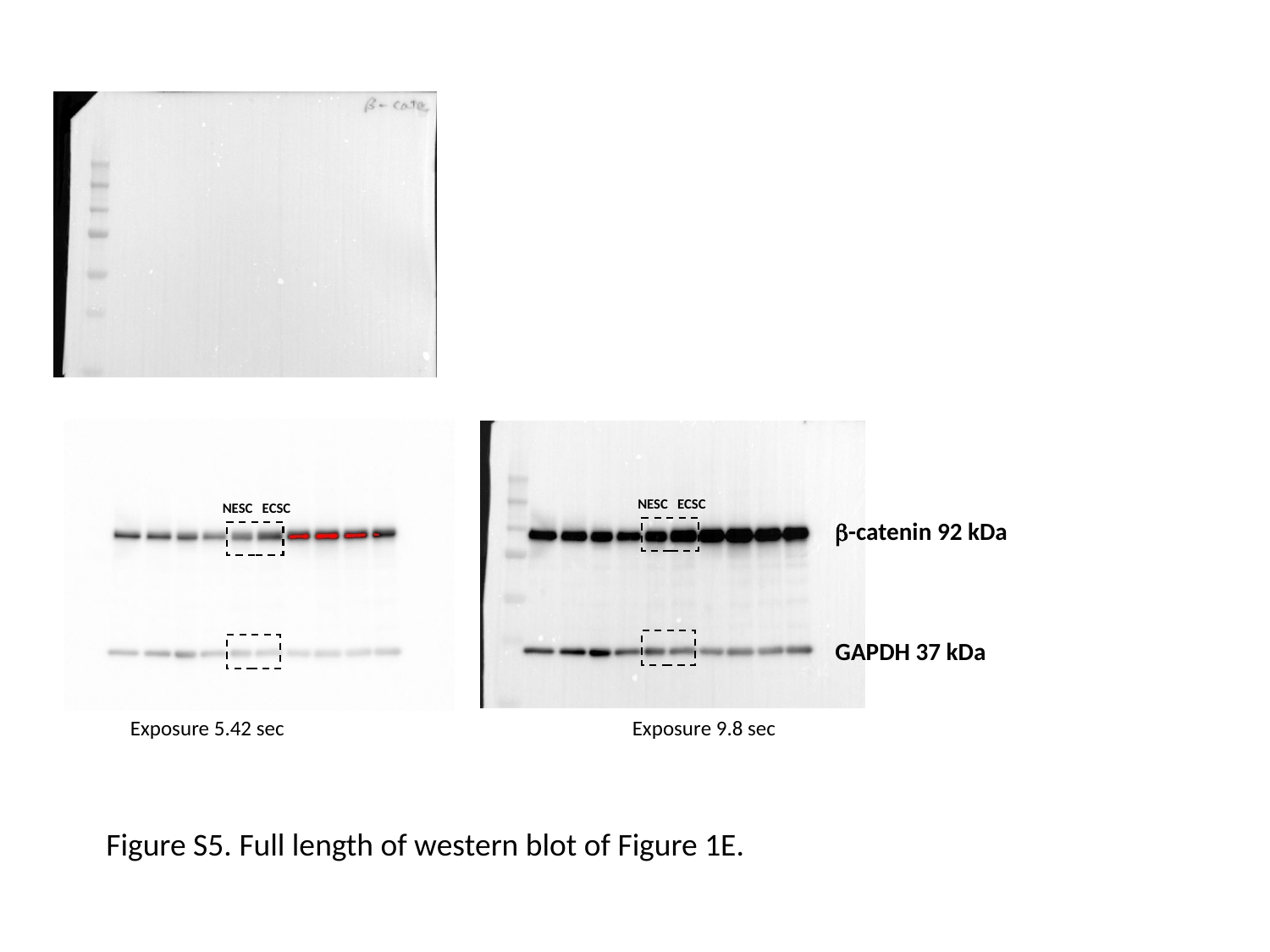

NESC ECSC
NESC ECSC
b-catenin 92 kDa
GAPDH 37 kDa
Exposure 5.42 sec
Exposure 9.8 sec
Figure S5. Full length of western blot of Figure 1E.

## Slide 2
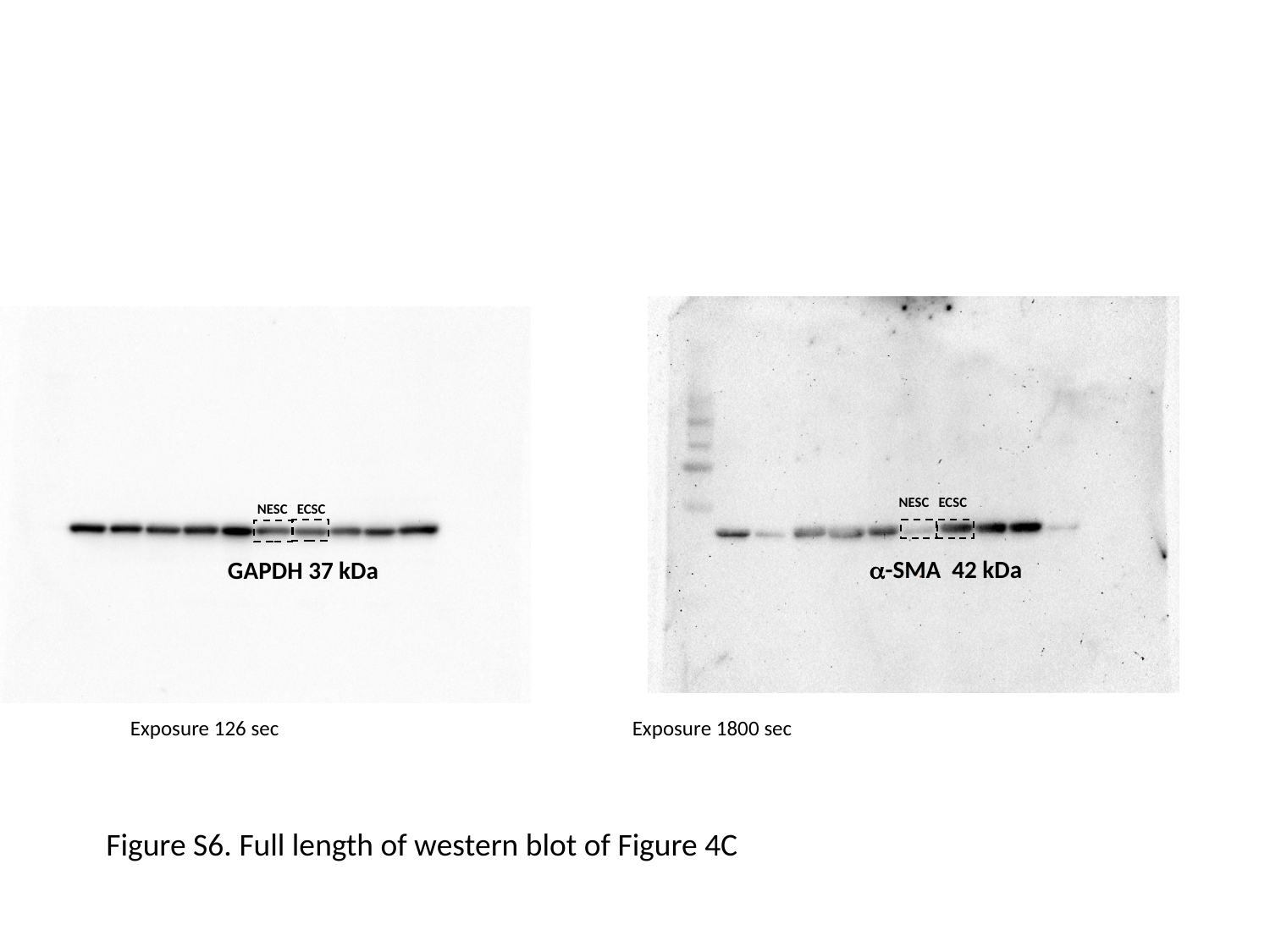

NESC ECSC
NESC ECSC
a-SMA 42 kDa
GAPDH 37 kDa
Exposure 126 sec
Exposure 1800 sec
Figure S6. Full length of western blot of Figure 4C

## Slide 3
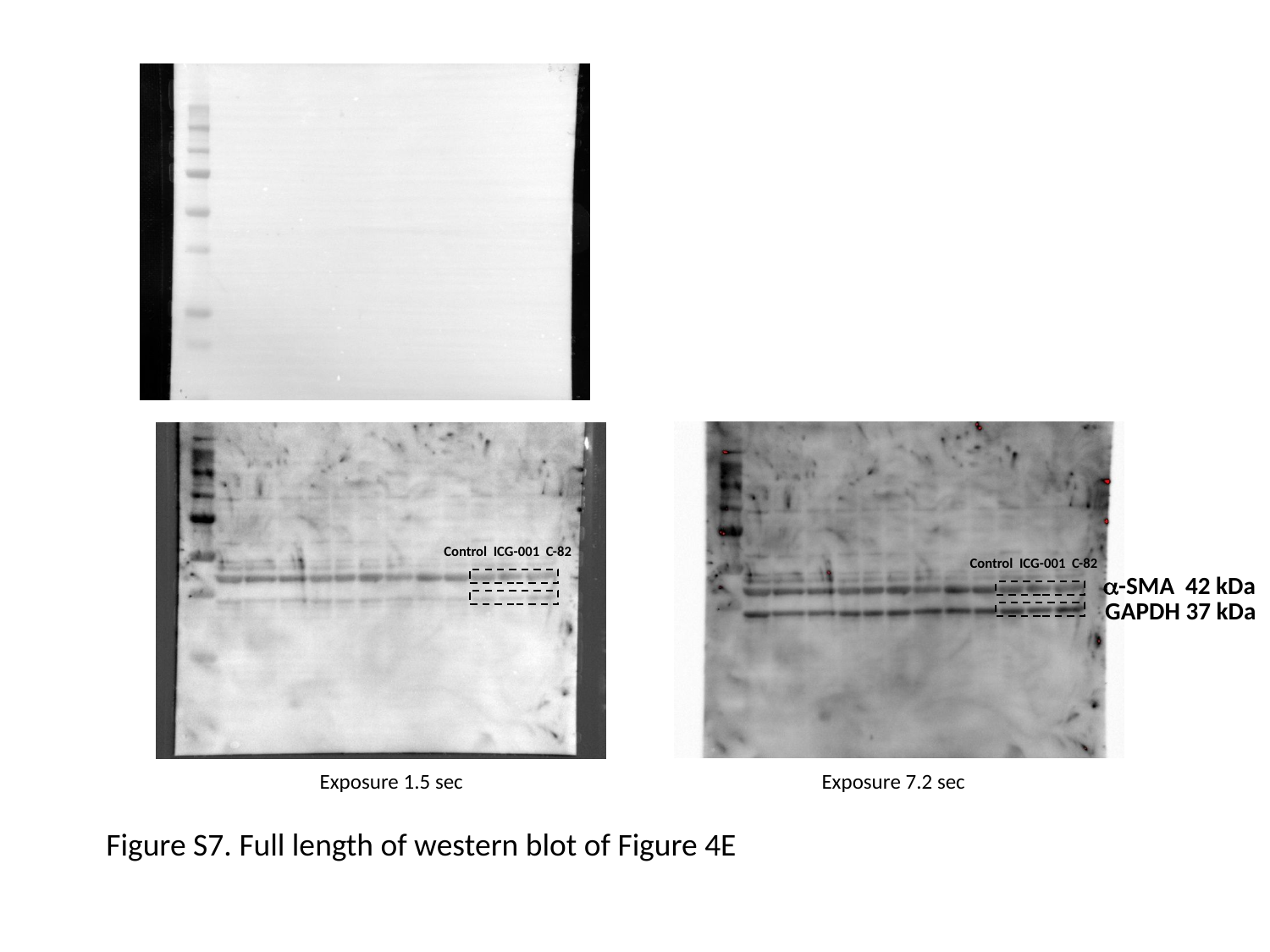

Control ICG-001 C-82
Control ICG-001 C-82
a-SMA 42 kDa
GAPDH 37 kDa
Exposure 1.5 sec
Exposure 7.2 sec
Figure S7. Full length of western blot of Figure 4E
